# A CNN-Based Method for Enhancing Boring Vibration with Time-Domain Convolution-Augmented Transformer

**DOI:** 10.3390/insects14070631

**Published:** 2023-07-13

**Authors:** Huarong Zhang, Juhu Li, Gaoyuan Cai, Zhibo Chen, Haiyan Zhang

**Affiliations:** 1 School of Information Science and Technology, Beijing Forestry University, Beijing 100083, China; huarong2000@bjfu.edu.cn (H.Z.);; 2 Engineering Research Center for Forestry-Oriented Intelligent Information Processing of National Forestry and Grassland Administration, Beijing 100083, China

**Keywords:** boring vibration, pest management, deep learning, neural network, attention mechanism

## Abstract

**Simple Summary:**

Trunk-boring insects have emerged as one of the most threatening forest pests globally, causing significant damage to forests. Certain groups of larvae reside within tree trunks without any observable external signs indicating their presence. This poses a significant challenge for pest detection. To tackle this issue, acoustic techniques are frequently utilized, which involve embedding a vibration probe into the tree trunk to capture the vibrations produced by larvae and using a model to distinguish whether the tree is infested. However, this approach requires the acquisition of the purest possible vibrations signal. Thus, to ensure accurate analysis, a noise suppression process is crucial, since the signals collected in real-world environments are often subjected to varying degrees of environmental noise interference. In this study, we employed artificial intelligence techniques to develop a boring vibration enhancement model. The training data utilized in this study comprise boring vibrations captured from trunk sections and typical environmental noise present in the trees’ habitat. The experimental results demonstrate that the enhancement method proposed by our model significantly boosts the performance of an established classification model. Overall, this study promotes the development of sustainable and efficient forestry protection approaches by improving the accuracy of pest detection.

**Abstract:**

Recording vibration signals induced by larvae activity in the trunk has proven to be an efficient method for detecting trunk-boring insects. However, the accuracy of the detection is often limited because the signals collected in real-world environments are heavily disrupted by environmental noises. To deal with this problem, we propose a deep-learning-based model that enhances trunk-boring vibration signals, incorporating an attention mechanism to optimize its performance. The training data utilized in this research consist of the boring vibrations of *Agrilus planipennis* larvae recorded within trunk sections, as well as various environmental noises that are typical of the natural habitats of trees. We mixed them at different signal-to-noise ratios (SNRs) to simulate the realistically collected sounds. The SNR of the enhanced boring vibrations can reach up to 17.84 dB after being enhanced by our model, and this model can restore the details of the vibration signals remarkably. Consequently, our model’s enhancement procedure led to a significant increase in accuracy for VGG16, a commonly used classification model. All results demonstrate the effectiveness of our approach for enhancing the detection of larvae using boring vibration signals.

## 1. Introduction

The world’s forests exert influence on climate through physical, chemical, and biological processes that impact planetary energy dynamics, the hydrological cycle, and atmospheric composition [1]. They also play a vital role in supporting human livelihoods by providing resources such as timber, food, and medicine. However, the sustainability of forests is being threatened by a variety of factors, including biotic factors such as insect pests and diseases and abiotic factors such as climate change and natural disasters, as well as anthropogenic factors such as deforestation, land-use change, and pollution [2]. Among all biotic factors, tree pests and pathogens are a major and increasing threat to the integrity of forest ecosystems [3]. They are the most pervasive and important agents of disturbance in North American forests, affecting an area almost 50 times larger than fires and with an economic impact nearly five times as great [4]. Moreover, with the development of globalization, the intensification of species invasion is becoming more and more severe, and this phenomenon will continue to worsen. The trunk-boring beetle is one of the most difficult to manage among forest pests. They tunnel and feed in the cambium layer of trees, which transports nutrients and water to the leaves [5]. This will lead to a gradual death of the tree due to insufficient nutrient uptake, and it is difficult to detect at an early stage. Therefore, early, accurate, and efficient detection of such pests is crucial for biological control and environmental protection.

To address the issue of pest infestations and prevent tree mortality, many studies have been conducted to develop techniques for early detection and treatment [6]. Various monitoring methods have been devised, including visual inspection, suction traps, and passive methods, such as baited traps using species-specific pheromones or blends and host volatiles to attract multiple species [7]. These manual monitoring methods require skilled operators to physically visit each observation location regularly, leading to consistently high labor costs and potentially resulting in low efficiency, slow response times, and inadequate sample sizes in some cases [8]. Therefore, the current research is primarily focused on developing techniques for monitoring using nonartificial methods. Acoustic technology has a long history of supporting insect pest managers with decision tools and providing information difficult to obtain about insect behavior, physiology, abundance, and distribution [9]. Al-Manie et al. [10] used an acoustic instrument for early discovery of the presence of a destructive insect pest, commonly known as the Red Date Palm Weevil (RDPW). Fiaboe et al. [11] demonstrated successful identification of insect larvae by recording and analyzing the sounds they produce while moving and feeding within trunk sections or fronds in a laboratory setting. Neethirajan et al. [12] investigated the benefits of utilizing acoustic technology for detecting insects in stored grains compared with the expensive and complex X-ray and NIR spectroscopy methods. Banga et al. [13] highlights that among various methods for detecting pests, including some novel technologies, the acoustic method is the key focus, which has good commercial potential. Mankin et al. [14] developed a portable insect detection system based on acoustic technology that assess the likelihood of insect infestations in bags of grain, flour, and other commodities that are stored in small holdings in developing countries, enabling rapid targeting of treatments. These studies have demonstrated the significance of acoustic technology in the field of insect pest detection. However, the process of signal acquisition is heavily influenced by environmental factors in the background. In practical applications, the acoustic monitoring of trunk borers is often conducted in an environment with unavoidable interference, especially environmental noise. The environmental noises may include natural sounds such as wind, rain, and animal vocalizations, as well as human-generated sounds such as nearby traffic, machinery, and human voices. The presence of these sounds poses challenges to accurate detection, as they can significantly degrade the quality of the collected boring vibration signals. It is crucial to note that the quality of the collected signals is closely related to the detection accuracy of models. Zhou et al. [15] proposed improved antinoise power-normalized cepstral coefficients (PNCCs) based on the wavelet package for detecting trunk borer vibrations. The detection accuracy was as high as 97% at 0 dB but decreased to 83% at −5 dB and further to 70% at −10 dB. A novel Fusion Fisher Vector (FFV) features are proposed in Ref. [16] for Acoustic Event Classification (AEC) in the meeting room environments. Its detection accuracy decreased by 3% and 7.7% compared with the detection accuracy in noisy conditions at 10 dB and 0 dB, respectively. Environmental noise remains a persistent challenge that hinders the accuracy of detection results and requires further attention for effective resolution.

The constant and rapid advancements in artificial intelligence technology and signal analysis have effectively bridged the gap between theoretical foundations and practical applications. It is now opportune to apply these cutting-edge technologies to mitigate the detrimental impacts of environmental noise on accurately identifying the vibrations of targeted insect species. In the existing research, the denoising methods of acoustic signals could be roughly categorized into two prominent families of approaches: traditional denoising methods and deep-learning-based denoising methods. Traditional methods such as spectral subtraction [17] and Wiener filtering [18] are based on the assumed statistical properties of noise signals and are limited in their effectiveness, because real-world noise is highly unpredictable and unstable. Deep learning, as an artificial intelligence technology, allows computational models that are composed of multiple processing layers to learn representations of data with multiple levels of abstraction [19]. These methods have dramatically improved the state-of-the-art in speech recognition, speech enhancement, object detection, and many other domains such as drug discovery and genomics. Liu et al. [20] proposed a deep-learning-based model to enhance the mixture of *Semanotus bifasciatus* larval feeding vibrations and various environmental noises. Shi et al. [5] introduced a waveform mapping-based approach for enhancement of trunk borers’ vibration signals also using a deep learning model. In their work, they proposed a structure called SRU++, which has demonstrated effectiveness in enhancing vibration signals. They synthesized noisy signals by mixing clean boring vibration signals with noise to simulate the signals collected in forested environments. Subsequently, the synthesized signals were input into the network for enhancement, and the enhanced signals were compared with the clean signals to demonstrate the effectiveness of the enhancement model. Nevertheless, the enhanced signals failed to achieve the desired SNR and exhibited notable distortion when compared with the clean signals. Building upon previous work, this study further investigates this direction and proposes a more effective model to address these issues. As demonstrated in the Results section, our model surpasses previous research in terms of both SNR and the fidelity of vibration signals. These latest studies took advantage of deep-learning-based speech enhancement. Speech enhancement aims to remove noise or interference from noisy speech in order to reconstruct the clean speech [21]. Since one probe is capable of detecting feeding sounds within a spherical region of a trunk [22], the resulting signals will be single-channel, and monaural speech enhancement is the proper technique for this situation.

In theory, deep-learning-based single-channel (monaural) speech enhancement methods can be divided into two categories according to the domain in which they perform. One is the time–frequency (T-F) domain, and the other is the time domain [23]. The time-domain approaches [24,25] predict a clean speech waveform directly from the corresponding noisy speech waveform, while T-F domain approaches [26] generally take the noisy spectral feature or the mask (e.g., Ideal Binary Mask [27], Ideal Ratio Mask [28], and complex Ideal Ratio Mask [29]). In T-F domain methods, the input speech signal is commonly transformed into time–frequency representations using the Short-Time Fourier Transform (STFT). Methods based on time domain and frequency domain have achieved excellent results in speech enhancement. Transformer [30] is a neural network architecture based on the attention mechanism. It is capable of processing input data in parallel, effectively solving the problem of long-term dependency and significantly reducing both training and inference time. Transformer is widely used in natural language processing [31] and automatic speech recognition [32] tasks due to its strong modeling and representation capabilities for sequence modeling. However, the use of contextual feature information in speech enhancement is different from that in natural language processing and other natural language processing tasks. In the context of speech enhancement tasks, contextual features refer to the contextual information related to the target speech signal, whereas in natural language processing tasks, contextual features typically refer to the information of surrounding words or sentences. Therefore, traditional Transformer neural networks do not perform well in speech enhancement. Convolution-augmented Transformer [33] is an improvement over the Transformer, which adopts local a self-attention mechanism to better capture the local dependencies within a sequence, thus making it more effective for long-sequence tasks, especially speech sequences. Recently, both Transformer and Conformer have been widely applied in the field of speech enhancement, and this study was also inspired by them.

The emerald ash borer (EAB), *Agrilus planipennis* Fairmaire (Coleoptera: Buprestidae), is an invasive pest that originated from Asia [34]. Its larvae burrow into the trees and create tunnels, which prevent the tree from receiving sufficient nutrients and water, ultimately resulting in the death of the ash trees. This study recorded the boring vibration of EAB and the corresponding environmental noise to construct our dataset. Inspired by the stated problems and previous works, we designed a time-domain model with Conformer called the Time-domain Conformer-based Enhancement Network for Vibration (T-CENV) to enhance EAB larval boring vibration. the T-CENV achieved excellent performance in noisy environments with different SNRs, providing a denoising scheme for acoustic detection and reducing the parameter size to a mere 6 million. This facilitates the direct deployment of the proposed denoising model to embedded devices.

This paper is organized as follows. In Section 2, we describe the composition of our dataset in detail. Section 3 presents the proposed framework, which is followed by the experimental process and result analysis in Section 4. Finally, some conclusions are drawn in Section 5.

## 2. Dataset Materials

### 2.1. Data Collection and Treatment

We used the same raw data as in a previous study [5], we selected and cut down a portion of the tree trunks from an ash forest, including those that were dying, alive, and already dead, located in Tongzhou District, Beijing, which had been infested with EAB larvae. To obtain the boring vibration signal conveniently, we cut the tree trunk into equal-length segments and removed all branches. After selecting, we acquired 6 trunk sections from alive trees, 4 from dying trees, and 2 from dead trees. Subsequently, we drilled the probe of a piezoelectric vibration sensor into the trunk sections to capture locomotion and feeding sounds generated by the EAB larvae. This sensor was jointly developed by Beijing Forestry University and Beihang University. We relocated all of the equipment and trunk segments to a relatively quiet idle lab to avoid interference from other noises. All vibration signals were gathered by the sensor and saved in WAV format on the computer using Audacity. The recording time for each trunk was one and a half hours per day. The audio was composed of many audio clips that were clipped and spliced together, because the vibration signals were intermittent. There was only one audio clip for each trunk that was either dying and dead because they barely have larvae activity, and most of the recordings contain only noise floor as instrumentation. To ensure that all vibrations were solely attributed to EAB larvae, we meticulously peeled the bark from all trunk segments after collection and accurately counted the number of EAB larvae under the supervision of forestry specialists.

After acquiring the raw signals, we conducted initial processing on the data. In order to enhance the robustness of our model, we applied distinct preprocessing methods to the dataset. We trimmed each clip’s beginning and end to eliminate noise from recording operations. After that, we meticulously examined the spectrogram to remove any sections of the clips that contained anomalous noises during the recording process, such as footsteps, raindrops, door closing sounds, sudden bursts emitted by instruments, etc. We also discarded the entire clip if it did not contain enough larval activity.

### 2.2. Environmental Noise Collection

In order to train the model, we employed a methodology similar to that used in the field of speech enhancement, where environmental noises and clean vibrations were combined to generate noisy boring vibrations. This approach created a dataset that closely simulates real-world scenarios. Therefore, we carefully selected five locations that were similar to the growth environment of the ash trees to collect noise. Four are located at Beijing Forestry University, and the other is in Olympic Forest Park. In addition, to ensure the data’s validity and sake of consistency, we employed identical equipment and techniques as those for collecting boring vibrations. We inserted the same probe into an ash trunk and used the same computer to store the signals. The collected environmental noise primarily comprised the rustling of leaves, birdsong, cicada chirping, footsteps, sough of the wind, and tire noise. Additionally, we discarded all the segments that did not contain noise to ensure high training efficiency.

### 2.3. Dataset Construction

In this study, all vibration signals were sampled at a high rate of 32 kHz. To facilitate model processing, we partitioned all recordings and environmental noises into a 3 s duration with a step length of 2 s. Adjacent signal segments overlapped by 1 s. After segmentation, we obtained 29,948 clean boring vibration signals and 426 environmental noise signals. In our dataset, 90% of the boring vibration and noise segments were divided into the training set, and the other 10% were designated as the test set. To intuitively observe the effect of each training epoch, we allocated 95% of the segments in the training set for training and 5% for validation.

**Figure 1:**
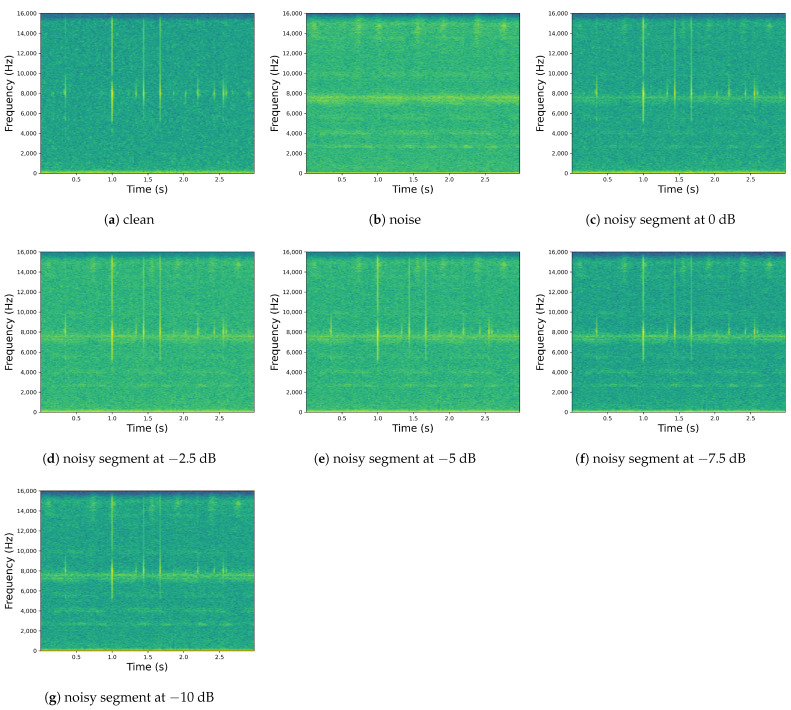
Spectrograms of boring vibration segments and environmental noise: (**a**) clean boring vibration spectrogram and (**b**) environmental noise spectrogram noisy segments at (**c**) 0 dB, (**d**) −2.5 dB, (**e**) −5 dB, (**f**) −7.5 dB, and (**g**) −10 dB.

To generate the training set, 26,953 clean boring vibrations were selected, and 383 environmental noise signals were, respectively, mixed with these clean signals according to 5 different SNRs (0 dB, −2.5 dB, −5 dB, −7.5 dB, and −10 dB), resulting in a total of noisy boring vibrations under 1915 different noisy environments. To generate the test set, the remaining 2995 clean boring vibrations were mixed with all 426 environmental noise signals at the same 5 SNRs (0 dB, −2.5 dB, −5 dB, −7.5 dB, and −10 dB) to create a total of noisy boring vibrations in 2130 different noisy environments. Consequently, our dataset includes 26,953 training examples, totaling about 22.5 h in duration, and 2995 testing examples, totaling about 2.5 h in duration. Compared with previous studies, our approach of mixing signals with different SNRs can better simulate real-world noise environments, as noise in natural environments is complex and diverse. Figure 1 demonstrates the clean boring vibration segment, the environmental noise segment, and the noisy segments after mixing boring vibration and noise at different SNR levels.

## 3. Methods

### 3.1. Problem Formulation

Enhancement of vibration signals is similar to that of speech signals, in which the aim is to estimate the source signals mixed on a single channel. Therefore, the noisy vibration signal can be represented as follows:

(1) y(t)=s(t)+d(t),

In Equation (1), y(t) represents the noisy boring vibration signals, s(t) represents the clean boring vibration signals, and d(t) represents the environmental noises. The task of this study is to estimate the clean boring vibration signals $\hat{s}\left(t\right)$ from y(t) such that the difference between $\hat{s}\left(t\right)$ and s(t) is minimized, which can be expressed as

(2) $$\min\left(dis\left(\hat{s}\left(t\right),s\left(t\right)\right)\right),$$

In Equation (2), dis measures the difference between $\hat{s}\left(t\right)$ and s(t).

### 3.2. Model Architecture

**Figure 2:**
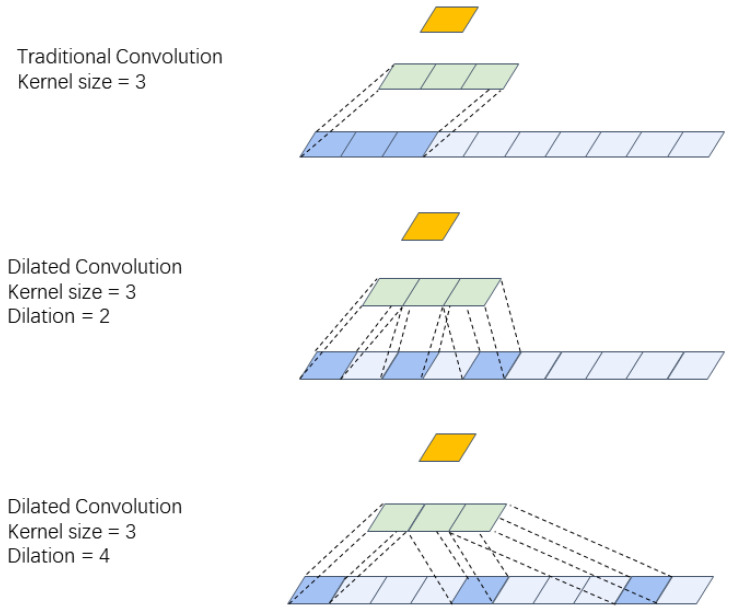
Traditional convolution uses a kernel size of 3, while dilated convolution uses a kernel size of 3 and a dilation of 2 and 4. The green block represents the convolution kernel, while the orange block represents the current output.

The Convolutional Encoder–Decoder (CED) network proposed in [35] consists of symmetric encoding layers and decoding layers. This encoder architecture is composed of several repetitions of a convolutional layer, batch normalization layer [36], max pooling layer, and LeakyReLU activation function [37]. This combination of layers has been shown to effectively extract features from input data and improve the performance of neural networks in various image and signal processing tasks. The convolutional layer is responsible for convolving the input data with learnable filters to extract important features. The batch normalization layer is used to normalize the feature maps generated by the convolutional layer, which helps to improve the convergence of the network during training. The max pooling layer is used to downsample the feature maps and reduce the spatial dimensions of the data, while the LeakyReLU activation function introduces nonlinearity to the network and helps to prevent the problem of vanishing gradients. Similar to the structure of the encoder, the decoder consists of repetitions of a convolution, batch normalization, and an upsampling layer. Typically, CED compresses the features along the encoder and then reconstructs the features along the decoder. However, the traditional CED architecture is mainly proposed for images, and speech is a kind of temporal information where the max pooling layer can destroy the information, leading to suboptimal performance. Therefore, in this study, we removed the max pooling layer in the encoder. In addition, we utilized dilated convolutions [38] as the primary convolution operation in the encoder, instead of traditional convolutions, and employed transposed convolutions [39] for upsampling in the decoder. Dilated convolution can increase the receptive field of a convolutional layer without increasing the number of parameters. In traditional convolution, the receptive field of the convolution kernel is fixed, while in dilated convolution, the receptive field of the convolution kernel can be expanded by increasing the dilation rate of the kernel, allowing for a larger range of information perception without increasing the number of model parameters. This method can enhance the model’s ability to capture temporal features over longer sequences [40]. Figure 2 illustrates the structure of convolution and dilated convolution. Transposed convolution, also known as deconvolution, is a powerful tool that can transform low-dimensional feature maps into high-dimensional feature maps. Compared with traditional interpolation methods, transposed convolution can preserve more contextual information, thereby improving the accuracy of the model.

**Figure 3:**
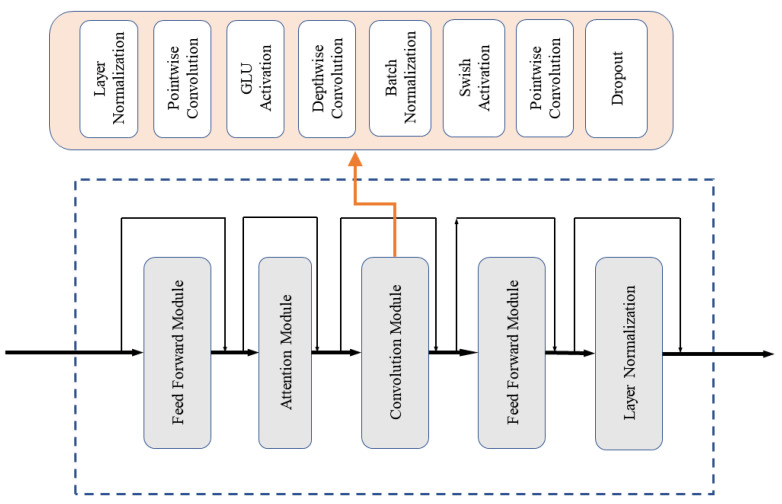
The structure of the T-Conformer.

Recurrent neural networks (RNNs) are a powerful model for sequential data [41], and the most prominent example of RNNs is the long short-term memory (LSTM) network. However, the training process of LSTM can become challenging due to the problem of vanishing or exploding gradients during the backpropagation process. Additionally, LSTM requires a large number of parameters to model, leading to high model complexity. Therefore, in this study, we proposed a time-domain Conformer (T-Conformer) structure. Figure 3 illustrates the structure of the T-Conformer, which comprises a convolutional module, an attention module, and two feed-forward modules, with skip connections between different modules. The convolution module contains a pointwise convolution with an expansion factor of 2 that projects the number of channels with a GLU activation layer, followed by a depthwise convolution. The depthwise convolution is followed by a batch normalization and then a swish activation layer. Compared with LSTM, the T-Conformer is capable of processing input sequences in parallel using self-attention, which significantly reduces both training and inference times. Additionally, pointwise convolution and depthwise convolution further enhance its processing efficiency.

**Figure 4:**
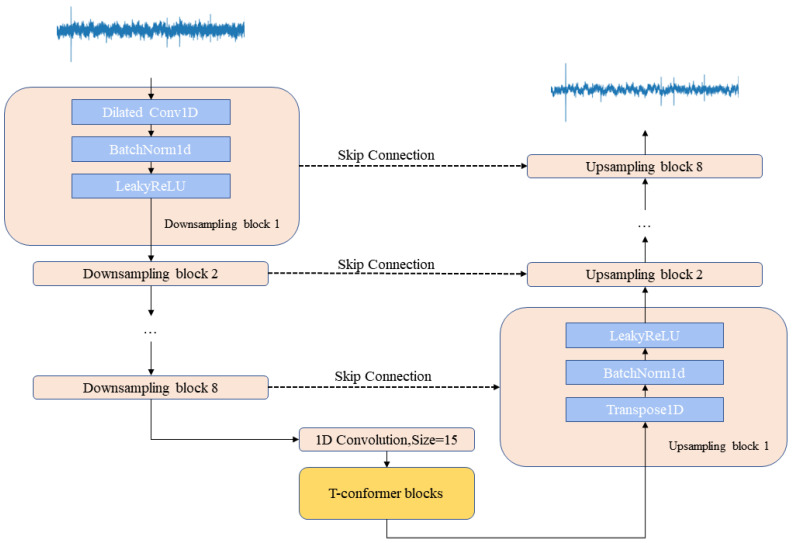
The structure of the T-CENV.

The T-CENV is composed of an encoder, a decoder, and two layers of T-Conformers. The encoder in the T-CENV model comprises eight downsampling layers, each composed of a dilated convolution layer, a batch normalization layer, and a LeakyReLU activation function. These layers are applied sequentially to the time-domain signal (raw waveform) of the boring vibration to obtain the corresponding latent representations. As the convolutional layers in the encoder can only extract local high-level features [42], these latent representations are then fed into the T-Conformer blocks, which capture local and global dependencies to model the sequence information. To correspond to the encoder, the decoder also has eight upsampling layers. Each upsampling layer includes a 1D transposed convolution layer followed by a batch normalization layer and a LeakyReLU activation function layer. The decoder reconstructs the time-domain boring vibration signal from a latent representation estimated through the T-Conformers. A skip connection connects the output of each encoder layer to the input of the corresponding decoder layer. Figure 4 illustrates the overall structure of the T-CENV. All convolutions have a kernel size of 15, a stride of 2, and a padding of 7. All transposed convolutions have a kernel size of 5, a stride of 2, and a padding of 2. All leakyReLU activation function layers have a 0.1 negative slope. The output channels of the encoder layers are 16, 32, 64, 64, 128, 128, 256, and 256, respectively. To maintain the shape of the output the same as the input, the number of input channels of the transposed convolution is twice the number of output channels of the corresponding encoding layer, and the number of output channels is the same as that of the previous encoding layer.

### 3.3. Loss Functions

We selected three loss functions, Mean Squared Error (MSE) loss, negative SNR loss, and L1 loss, based on previous studies that have shown good performance in enhancing boring vibrations. They are mathematically expressed as follows:

(3) $$MSE=\frac{1}{N}\sum_{i=0}^{N-1}{\left(y_{i}-{\hat{y}}_{i}\right)}^{2}$$

(4) $$negativ\mathrm{e-SNR}=-10\log_{10}\frac{\sum_{i=0}^{N-1}{y_{i}}^{2}}{\sum_{i=0}^{N-1}{\left(y_{i}-{\hat{y}}_{i}\right)}^{2}}$$

(5) $$L1=\frac{1}{N}\sum_{i=0}^{N-1}\left|y_{i}-{\hat{y}}_{i}\right|$$

In Equations (3)–(5), *i* refers to the *i*-th sampling point, and *y* and $\hat{y}$ represent the clean signal and enhanced signal, respectively. Our experimental results presented in the next section demonstrate that using Negative-SNR as the loss function achieves the best model performance among the tested loss functions.

### 3.4. Evaluation Metrics

In this study, we assessed our model’s performance using three widely adopted objective metrics: the signal-to-noise ratio (SNR), the segmental signal-to-noise ratio (SegSNR), and the log-likelihood ratio (LLR). We refrained from utilizing popular speech enhancement evaluation metrics, such as Perceptual Evaluation of Speech Quality (PESQ) [43], Short-Time Objective Intelligibility (STOI) [44], etc., owing to their speech-specific design. Deploying them to boring vibration signals would be inappropriate. Hence, we employed the same evaluation metrics as in prior boring vibration signals research. SNR represents the ratio of energy in clean boring vibration sound to that of noise. SegSNR is an average SNR value computed per frame, and LLR represents the ratio of the energies of the prediction residuals of the enhanced and clean signals. The equations for them are provided as follows:

(6) $$SNR=10\log_{10}\frac{\sum_{i=0}^{N-1}{y_{i}}^{2}}{\sum_{i=0}^{N-1}{\left(y_{i}-{\hat{y}}_{i}\right)}^{2}}$$

(7) $$SegSNR=\frac{10}{F}\sum_{n=0}^{F-1}\log_{10}\frac{\sum_{i=L_{n}}^{L_{n}+L-1}{y_{i}}^{2}}{\sum_{i=L_{n}}^{L_{n}+L-1}{\left(y_{i}-{\hat{y}}_{i}\right)}^{2}}$$

In Equations (6) and (7), *y* denotes the clean vibration signal, and $\hat{y}$ denotes the enhanced signal. *N* represents the number of samples. *F* is the number of frames in a segment of signal, and *L* is the frame length.

(8) $$LLR\left(a_{x},{\bar{a}}_{\hat{x}}\right)=\log\frac{{\bar{a}}_{\hat{x}}^{T}R_{x}{\bar{a}}_{\hat{x}}}{a_{x}^{T}R_{x}a_{x}}$$

In Equation (8), $a_{x}^{T}$ are the LPC coefficients of the clean signal, ${\bar{a}}_{\hat{x}}^{T}$ are the coefficients of the enhanced signal, and $R_{x}$ is the autocorrelation matrix of the clean signal [5].

### 3.5. Implementation Details

In order to evaluate the effectiveness of our proposed T-CENV model and demonstrate the superiority of using the Negative-SNR loss function, we conducted comparative experiments using both the T-CENV-MSE and T-CENV-L1. Additionally, we conducted ablation experiments to demonstrate the effectiveness of our proposed T-Conformer module. All models were developed using PyTorch [45], a Python-based scientific computing library designed to offer efficient multidimensional tensor operations and automatic differentiation capabilities. The sampling rate used is 32 kHz. For the convenience of training, we split all the noisy boring vibrations into equally sized 3 s frames, and the step size was set to 2 s. We adopted the Adam optimizer [46] with an initial learning rate of 0.001, which is halved if the loss value does not decrease in the validation set in three consecutive epochs. The early stopping strategy is also employed in our model training. If the loss value does not decrease in 6 consecutive epochs on the validation split, the training stage is stopped. In addition, to speed up the training process, we also employed the method of multi-GPU parallel training. The hardware platform for our experiments consisted of a workstation equipped with an Intel Xeon Gold 5120 processor (192 GB memory) and two NVIDIA T4 Tensor Core GPUs (16 GB*2 graphic memory), as well as another workstation with an AMD R7-5800H processor (16 GB memory) and an NVIDIA GeForce RTX 3060 laptop GPU (6 GB graphic memory).

## 4. Results

The Average SNR, SegSNR, and LLR of the T-CENV using different loss functions are shown in Table 1. In this experiment, we used the same two-layer T-Conformer T-CENV, with the only difference being the loss function used. To comprehensively evaluate the performance of the loss functions, we also recorded the number of epochs during training.

By comparing the data in the table, it can be observed that training with L1 as the loss function is the fastest, and it achieves performance similar to MSE in Average SNR and Average SegSNR. However, the LLR metric is not as good. The Negative-SNR loss function achieved the best performance in terms of Average SNR, Average SegSNR, and Average LLR. Moreover, it requires 17 fewer training epochs compared with using MSE as the loss function. Therefore, there is no reason for us not to choose Negative-SNR as the loss function for training our model.

After determining the optimal loss function, we further examined the impact of the proposed T-Conformer and compared its performance with LSTM, as presented in Table 2.

After comparing the SNR, SegSNR, and LLR of the noisy vibration signals and the enhanced vibration signals generated by our proposed models, we found that the proposed models based on the T-ENV architecture showed enhancement effects to varying degrees. This result demonstrates that the use of an encoder–decoder structure for vibration signal enhancement is reasonable and effective. Then, we used the T-ENV model with only the encoder–decoder structure as the baseline. In addition to LSTM, we also introduced the Bidirectional Long Short-Term Memory Network (BiLSTM) [47] for comparative experiment. Unlike LSTM, the BiLSTM considers both past and future context information at each time step. It consists of two independent LSTM layers, one processing the input sequence in the forward direction and the other in the backward direction. The T-BiLSTM-ENV is composed of two layers of BiLSTM, while the remaining components are the same as T-LSTM-ENV. Comparing the results of T-LSTM-ENV and T-BiLSTM-ENV reveals that BiLSTM exhibits improved performance compared with LSTM. Specifically, the SegSNR metric shows an improvement of 11.11%. However, both LSTM and BiLSTM do not perform satisfactorily in terms of the LLR metric. By comparing the metrics of T-LSTM-ENV, T-BiLSTM-CENV, T-CENV, and T-ENV, we can observe that our proposed T-Conformer structure achieves the best performance. Theoretically, within a certain range, the more T-Conformer layers, the better the performance. Therefore, we conducted experiments on the number of T-Conformer layers and found that the best performance was achieved when the number of layers was two. When the number of layers reached 3, the enhancement effect began to decrease, and the number of parameters increased to 9.6 M. When the number of layers reached 4, the number of parameters increased to 11.4 M, and the enhancement effect further decreased.

The noise in real forest environments is diverse and highly unstable. To validate the robustness of our proposed model, we conducted experiments on test data with different SNRs, and selected T-LSTM-ENV and T-BiLSTM-ENV as the baseline models. To better showcase the comparative results, we selected SNR as the evaluation metric for this experiment. The experimental results are presented in Table 3. Through the observation of the results in Table 3, it can be concluded that the proposed T-CENV model outperforms T-LSTM-ENV and T-BiLSTM-ENV across various SNR conditions. The enhancement performance of T-CENV is 44.10% higher than that of T-LSTM-ENV in 0 dB.

**Figure 5:**
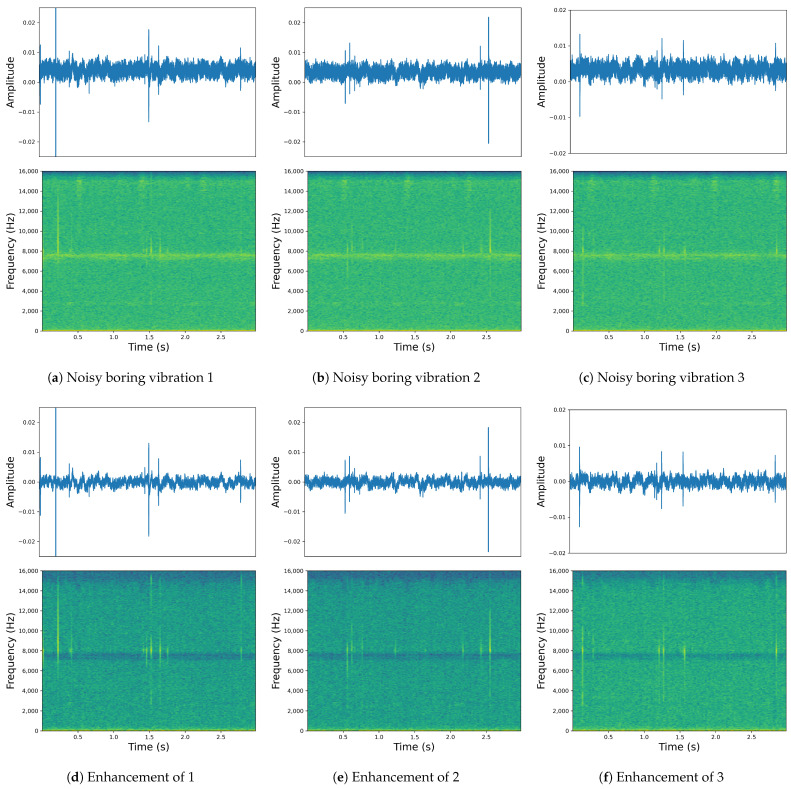
Comparison of the waveforms and spectrograms before and after enhancement using the T-CENV: The top row (**a**–**c**) displays the original signals, while the bottom row (**d**–**f**) shows the signals after T-CENV processing. The subfigures in the top row correspond to their counterparts in the bottom row, where (**a**) corresponds to (**d**), (**b**) to (**e**), and (**c**) to (**f**).

In summary, the T-CENV with a two-layer T-Conformer achieved the best enhancement performance, and the number of parameters was also relatively low. From the perspective of the enhanced vibration signal’s frequency spectra and waveforms, our model exhibits a very high level of detail recovery, which is crucial for the development of pest detection and prevention technologies using collected insect vibration signals. Figure 5 shows the partial waveform and spectrogram before and after enhancement using the T-CENV.

**Figure 6:**
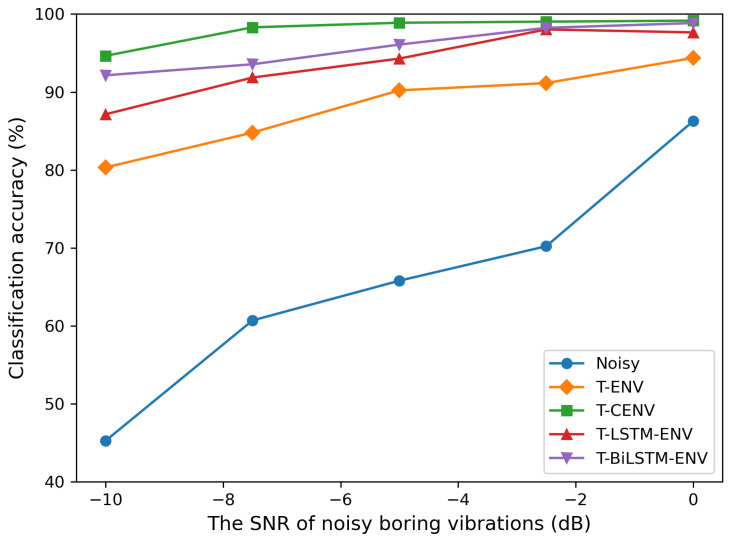
Comparison of classification accuracy on noisy segments, segments enhanced by T-ENV, T-CENV, T-LSTM-ENV, and T-BiLSTM-ENV.

As with previous studies, we also evaluated the detection accuracy of the well-known classification model VGG16 [48] on both noisy boring vibrations and vibrations enhanced by our model. The dataset used for training was also composed of the noisy and clean segments described in Section 2. The dataset was composed of two distinct categories: infested and uninfested. The infested class comprised 3428 noisy boring vibration segments and 3428 clean boring vibration segments. For the uninfested class, a total of 3195 noise segments and 2831 segments from dead trees were utilized. They were tested on a set of 1290 noisy boring vibration segments consisting of different signal-to-noise ratios (−10 dB, −7.5 dB, −5 dB, −2.5 dB, and 0 dB). The classification results for different kinds of data are shown in Figure 6. It was observed that as the SNR decreased, the classification accuracy of noisy boring vibrations decreased significantly, and the classification accuracy of enhanced signals obtained by different methods improved to different degrees. The segments enhanced by the T-CENV achieved the best classification results, suggesting that the T-CENV is more effective in removing distracting noise and enhancing the features of feeding sounds.

## 5. Discussion

In this study, we proposed a novel T-CENV model for enhancing boring vibration signals. This model is based on the encoder–decoder structure and uses the T-Conformer as the basic unit. The T-Conformer is a novel structure that combines the advantages of Transformer and a CNN. It can effectively capture the local and global dependencies of the input sequence. We compared various loss functions, and the experimental results indicate that employing Negative-SNR as the loss function in this study is appropriate. The utilization of Negative-SNR as the loss function effectively guides the training process of the model towards minimizing noise components in the signals. This promotes the improvement of signal clarity and quality. Although training with L1 as the loss function achieved the fastest training speed, it produced unsatisfactory performance. Furthermore, we performed a comprehensive performance comparison between the T-Conformer, LSTM, and BiLSTM models, revealing that the T-Conformer consistently outperforms both LSTM and BiLSTM in terms of performance. Additionally, we conducted a detailed analysis of models with different numbers of T-Conformer layers and identified the model that exhibited superior performance. In addition, the T-CENV model also achieved the best classification results on noisy boring vibration segments. All results demonstrate that the proposed T-CENV can effectively enhance boring vibration signals and has great potential for application in pest detection systems.

**Figure 7:**
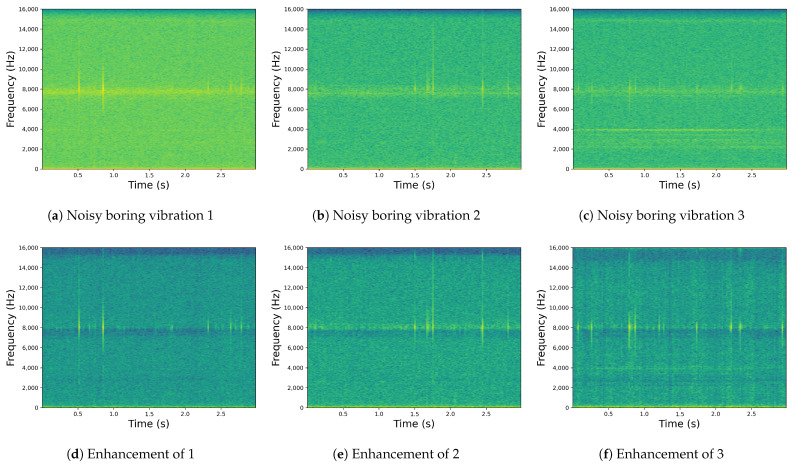

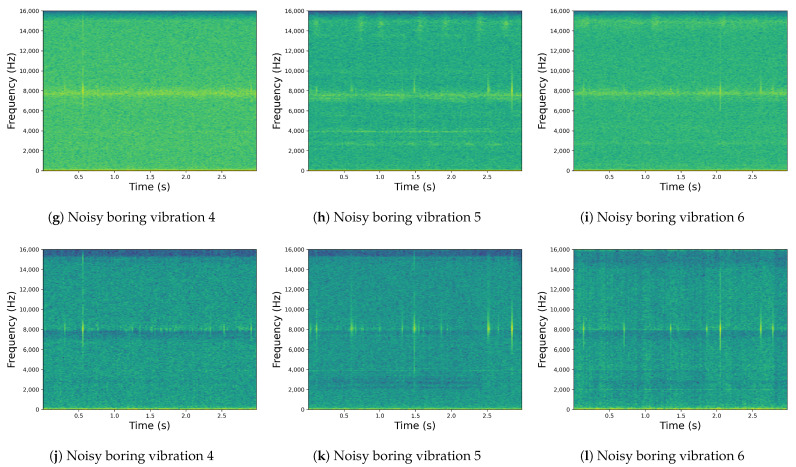
Comparison of the spectrograms before and after enhancement using the T-CENV: The top row (**a**–**c**,**g**–**i**) displays the signals that were directly recorded in habitats of the larval host plant, while the bottom row (**d**–**f**,**j**–**l**) shows the signals after T-CENV processing. The subfigures in the top row correspond to their counterparts in the bottom row, where (**a**) corresponds to (**d**), (**b**) to (**e**), (**c**) to (**f**), (**g**) to (**j**), (**h**) to (**k**), and (**i**) to (**l**).

To evaluate the enhancement performance of T-CENV under real conditions, we conducted tests using noisy vibration signals that directly recorded in habitats of the larval host plant. These signals were collected from an infested ash forest in Shunyi District, Beijing. The data segmentation method described in Section 2 was applied to ensure consistency in the evaluation process. Additionally, all vibration signals were sampled at a frequency of 32 kHz too. However, obtaining corresponding clean vibration signals in real-world environments is unattainable; so, it is infeasible to calculate objective metrics such as SNR, SegSNR, and LLR. Therefore, we selected a part of the representative enhanced results, as shown in Figure 7. The spectrograms clearly demonstrate the favorable enhancement effect of our model on real-world data. But there are still some challenges that need to be addressed. Firstly, the parameter count of the model still does not meet the requirements for embedded devices, necessitating further compression. In future research, we aim to explore model compression techniques to optimize the model. Secondly, the current dataset is still limited in terms of its diversity. Future research efforts will encompass the collection of vibration signals from a broader range of diverse environments to reinforce the training of the model. This endeavor will facilitate the model in attaining superior enhancement performance in real-world applications. Furthermore, continued research and development efforts are necessary to develop early warning system prototypes for trunk-boring beetles in both forested areas and urban environments. These innovative systems hold the potential to substantially reduce manual labor costs associated with pest control and management. This is also an area we aim to focus on and improve in our future research endeavors.

## 6. Conclusions

In modern forest management and pest control systems, it is a widely used and highly effective method to embed piezoelectric vibration sensors in tree trunks to collect activity information of pest larvae. However, in actual forest environments, signals collected are often susceptible to interference from other environmental sounds, which are typically uncontrollable. These sounds are recorded together with the desired pest activity sounds, which poses great challenges for the subsequent pest detection programs. Therefore, an enhancement method specifically for boring vibration signals is urgently needed. In this study, we conducted recordings of both the activity sounds of EAB larvae in tree segments and the environmental sounds present in actual forests. We carefully screened and segmented the recordings and subsequently mixed them at varying SNRs to simulate the signals typically collected in real-world environments. We designed the T-CENV using modern artificial intelligence techniques for enhancing trunk-boring vibration signals. In the T-CENV, the high-dimensional features of the input temporal vibration signals are first extracted using the encoder. Then, the decoder is employed to reconstruct the extracted features to obtain the enhanced vibration signals. To achieve the optimal performance of our model, we designed a T-Conformer architecture to enhance the model’s ability to extract both local and global information. The results demonstrate that the T-CENV had a strong ability to model the temporal features of boring vibrations and achieved excellent performance on the test set. Compared with networks using LSTM, the T-CENV achieved an improvement of approximately 44% in enhancing the signals. All the results indicate that our proposed T-CENV can effectively enhance the collected signals, and the SNR of the T-CENV-enhanced signals can reach up to 17.84 dB in complex noisy environments. Compared with previous studies, the T-CENV also can restore the details of the signal to a high degree, as evidenced by the enhanced spectrogram of the signal. This will lower the difficulty of developing pest detection systems and has significant value for forest pest detection.

## Figures and Tables

**Table 1:** Comparison of the enhancement performance of our model with different loss functions.

Loss Functions	Average SNR (dB)	Average SegSNR (dB)	Average LLR	Epochs
L1	12.23	11.50	0.5356	**10**
MSE	12.32	11.77	0.2257	52
Negative-SNR	**14.37**	**13.17**	**0.1638**	35

**Table 2:** Comparison of the enhancement performance between LSTM and models with different numbers of T-Conformer layers.

Models	Param. (M)	Average SNR (dB)	Average SegSNR (dB)	Average LLR
Noisy	-	−5.14	−5.96	−6.6067
T-ENV	**4.23**	10.57	9.89	0.1978
T-LSTM-ENV	5.28	10.99	9.99	0.4534
T-BiLSTM-ENV	6.99	12.17	11.10	0.5216
T-CENV × 1	6.02	14.03	12.83	0.3245
T-CENV × 2	7.81	**14.37**	**13.17**	**0.1638**
T-CENV × 3	9.60	13.66	12.47	0.3398
T-CENV × 4	11.04	11.01	10.06	0.6883

**Table 3:** The enhancement performance comparison of T-CENV and T-LSTM-ENV under different SNR conditions.

Models	0 dB	−2.5 dB	−5 dB	−7.5 dB	−10 dB
T-LSTM-ENV	12.38	11.88	11.18	10.27	9.24
T-BiLSTM-ENV	13.30	12.88	12.80	11.64	10.21
T-CENV	**17.84**	**16.12**	**14.27**	**12.58**	**11.05**

## Data Availability

The data presented in this study are available on request from the corresponding author.
